# Outcomes of valve-sparing aortic root replacement in patients with bicuspid aortic valve and tricuspid aortic valve: a systematic review and meta-analysis

**DOI:** 10.1186/s13019-023-02329-8

**Published:** 2023-07-03

**Authors:** Yiding Zuo, Ruixi Tan, Chaoyi Qin

**Affiliations:** 1 Department of Anesthesia, West China Hospital, Sichuan University, Chengdu, 610041 China; 2 Department of Cardiovascular Surgery and Cardiovascular Surgery Research Laboratory, West China Hospital, Sichuan University, 37th Guoxue Road, Chengdu, 610041 China

**Keywords:** Valve-sparing aortic root replacement, Reimplantation, Remodeling, Bicuspid aortic valve, Tricuspid aortic valve

## Abstract

**Background:**

Valve-sparing aortic root replacement (VSARR) is a safe and effective surgical procedure to treat aortic root aneurysm. This meta-analysis aimed to investigate how this procedure might differ in patients with bicuspid aortic valve (BAV) and tricuspid aortic valve (TAV).

**Design:**

Meta-analysis with meta-regression and systematic review.

**Setting:**

Systematic search in the following databases: PubMed, Cochrane Central Register of Controlled Trials, and Embase.

**Interventions:**

All observational studies of VSARR in patients with BAV or TAV were included in our study. Studies were included without any restrictions on language or publication date. A trial sequential analysis and a post-hoc meta-regression was performed on the main outcomes.

**Result:**

Eleven articles met the inclusion criteria. A total of 1138 patients in BAV group, and 2125 patients in TAV group. No significant differences in gender and age were observed between BAV and TAV patients. BAV and TAV patients showed no differences in in-hospital mortality rate [0.00% vs. 1.93%; RR (95% CI) 0.33 (0.09, 1.26), I^2^ = 0%, *P* = 0.11] and the rate of in-hospital reoperation [5.64% vs. 5.99%; RR (95% CI) 1.01(0.59, 1.73), I^2^ = 33%, *P* = 0.98]. The overall long-term mortality rate of BAV patients was better than that of TAV patients [1.63% vs. 8.15%; RR (95% CI) 0.34 (0.13, 0.86), I^2^ = 0%, *P* = 0.02]. During the follow-up observation period, patients in TAV group showed small but no statistic advantage in 3-year, 5-year, and over 10-year incidences of reintervention. Regarding the secondary endpoints, the two groups showed similar aortic cross-clamping time and total cardiopulmonary bypass time.

**Conclusion:**

The VSARR techniques yielded similar clinical outcomes in both BAV and TAV patients. Although patients with BAV might have a higher incidence of reinterventions after initial VSARR, it is still a safe and effective approach to treat aortic root dilation with or without aortic valve insufficiency. TAV patients showed small but no statistic advantage in long-term (over 10 years) reintervention rate, which means, patients with BAV may face a higher risk of reintervention in the clinic.

## Introduction

The traditional treatment for aortic root dilation is composite valve conduit, which is also known as the Bentall procedure. Although the Bentall procedure shows excellent long-term clinical results, the use of a bioprosthesis or a mechanical valve in this procedure is accompanied by some prosthesis- and coagulation-related complications [1]. The application of this technique to patients with a morphologically preserved native aortic valve has been debated [2]. An alternative approach for such patients is valve-sparing aortic root replacement (VSARR) surgery, which is performed by two different techniques: remodeling (Yacoub) and reimplantation (David) [3]. Both techniques have shown good mid-term and long-term clinical results [4, 5].

The remodeling technique physiologically preserves the aortic root. Remarkable mid-term and long-term outcomes have been reported for this technique, especially after combination with aortic ring annuloplasty [6]. The reimplantation technique was developed in 1989 by Dr. Tirone E. David and has undergone several modifications in the past two decades, leading to stabilization of the aortic annulus and excellent long-term results [7].

These good results were, however, observed largely in patients with tricuspid aortic valve (TAV). There are only limited data regarding the outcome of this technique in patients with bicuspid aortic valve (BAV). Therefore, the present meta-analysis aimed to evaluate the short-term and long-term clinical outcomes in TAV versus BAV patients who underwent VSARR.

## Methods

### Search strategy

We followed a scoring system based on a checklist derived from the criteria recommended by ROBSIN-I and PRISMA (Preferred Reporting Items for Systematic Reviews and Meta-Analyses) guidelines to assess the quality of trials included in the meta-analysis. We searched three major electronic databases, namely Medline [PubMed], Cochrane, and Embase, for all published articles that compared reimplantation in BAV and TAV patients, without any restrictions for language and publication date to make the literature retrieval more comprehensive. The search was conducted using the keywords “reimplantation” and “bicuspid” as the main search terms and “David procedure,” “Yacoub,” “valve-sparing,” and “remodeling” as complementary search terms. The specific main search formula was as follows: ((("Replantation"[Mesh]) OR (((((((Replantations [Title/Abstract]) OR (Surgical Replantation [Title/Abstract])) OR (Replantation, Surgical [Title/Abstract])) OR (Surgical Replantations [Title/Abstract])) OR (Replantation, Surgical [Title/Abstract])) OR (Reimplantation [Title/Abstract])) OR (Reimplantations [Title/Abstract]))))) AND ((bicuspid [Title/Abstract]) OR (bicuspids [Title/Abstract])).

### Inclusion and exclusion criteria

Two investigators independently assessed and screened the published data. No differences were noted in the assessment and screening procedures between these investigators. Studies with two arms comparing both BAV and TAV reimplantations were included in the meta-analysis, and observational studies on single-arm BAV and TAV reimplantations were excluded.

The screened studies were assessed with regard to control for confounders, measurement of exposure, and completeness of follow-up to ensure quality. A scoring system based on a checklist derived from the criteria recommended by the QUOROM (The Quality of Reporting of Meta-analyses) and PRISMA guidelines was followed to assess the quality of the trials included in this meta-analysis [8, 9].

### Data extraction and outcomes

Data regarding patient characteristics, study design, and clinical outcomes were extracted from the included studies. The primary outcomes of interest were in-hospital mortality, perioperative reoperation, > 1-year mortality, and mid-term and long-term freedom from reintervention. The secondary outcomes were interoperation ascending aortic cross-clamp time (ACx), cardiopulmonary bypass (CPB) time, and performing or not performing coronary artery bypass graft (CABG).

### Statistical analysis

Once the data were extracted, the ROBSIN-I tool was used to analyze and assess the bias in the selected studies [10]. All analyses were conducted using Review Manager software version 5.4.

## Results

### Baseline characteristics

**Fig. 1:**
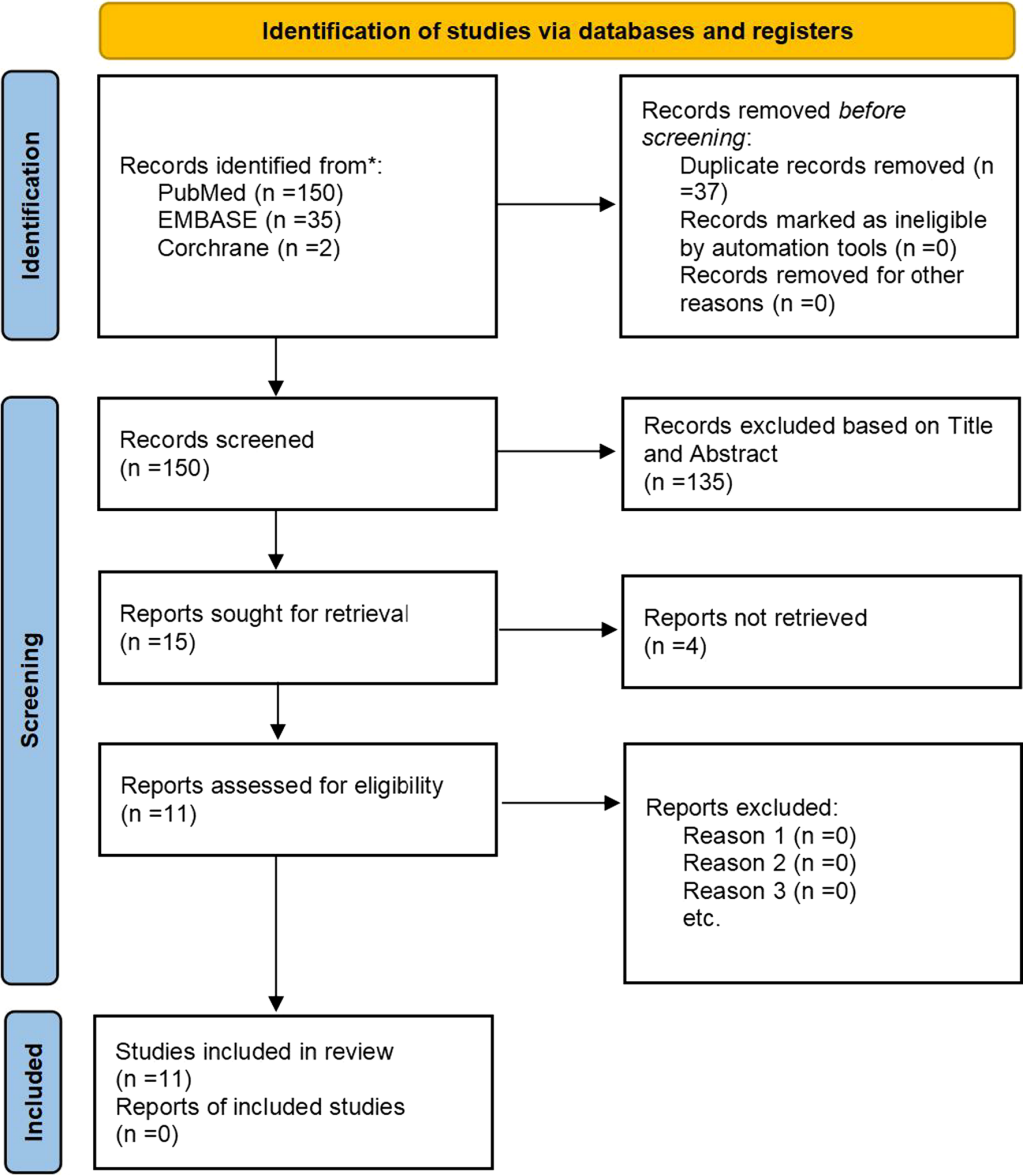
Selection algorithm

The initial search result yielded 150 nonduplicated articles that were screened by title and abstract. After applying inclusion and exclusion criteria, only 11 of the 150 studies met the final criteria and were included in the subsequent meta-analysis (Fig. 1).

**Fig. 2:**
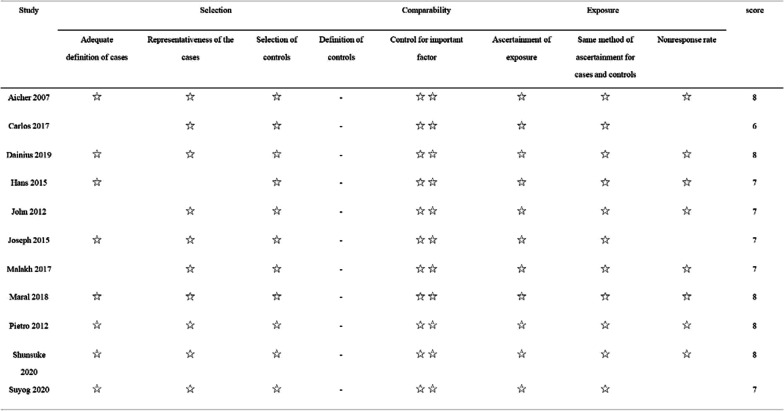
NOS quality evaluation form

A total of 3263 patients recruited in the 11 eligible articles were included in this analysis [2, 6, 11–19]. Of these patients, 1138 patients (43.9%) had BAV, and 2125 patients (65.1%) had TAV. No significant differences in age and gender were observed between the two patient populations (Table 1; Fig. 2).

**Table 1:** Summary of study characteristics

Author	BAV (N)	TAV (N)	Age (years)		*P*	Gender (male, %)		*P*
			BAV	TAV		BAV	TAV	
Aicher 2007	81	193	52 ± 12	62 ± 15	< 0.001	69, 85.2%	132, 68.4%	0.004
Carlos 2017	57	103	46.0 ± 11.8	57.5 ± 17.8	0.001	57, 100%	89, 88.1%	0.005
Dainius 2019	29	63	42.4 ± 12	55.3 ± 14.9	0.001	56, 88.9%	27, 93.1%	0.5
Hans 2015	290	431	54 ± 15			–	–	
John 2012	63	170	43 ± 12	36 ± 13	< 0.001	50, 79.4%	115, 67.6%	0.08
Joseph 2015	40	89	46 ± 12	45 ± 15	0.7	35, 87.5%	63, 70.8%	0.3
Malakh 2017	24	173	40(30–47)	49(35–62)	0.0051	21, 87.5%	123, 71.1%	0.72
Maral 2018	45	135	40 ± 13	41 ± 14	0.93	121, 89.6%	39, 86.7%	0.59
Pietro 2012	24	108	–	–		–	–	
Shunsuke 2020	414	589	–	–		–	–	
Suyog 2020	71	71	48 ± 12	49 ± 12		57, 80.3%	52, 73.2%	

Author	Country	Study period	Follow-up duration (years)
(a)			
Aicher 2007	Germany	October 1995–October 2006	10
Carlos 2017	Spain	March 2004–December 2015	5
Dainius 2019	Lithuania	April 2004–October 2016	10
Hans 2015	Spain	October 1995–December 2013	15
John 2012	America	1993–2009	10
Joseph 2015	America	2004–2013	5
Malakh 2017	Germany	1993–2015	8.7
Maral 2018	Canada	1988–2012	8.2
Pietro 2012	Italy	2002–2011	5
Shunsuke 2020	Germany	1995–2018	15
Suyog 2020	America	January 2002–July 2017	8

Author	BAV	TAV	Preoperative degree of AR				LV EF (%)		Marfan		Aortic dissection	
	(N)	(N)	I	II	III	IV	BAV	TAV	BAV	TAV	BAV	TAV
(b)												
Aicher 2007	81	193	71	64	116	23	–	–	0	5	6	40
Carlos 2017	57	103	224	37	3	3	85.4% EF ≥ 55%		107		7	
Dainius 2019	29	63	5	47	38	2	53.6 ± 7.5	48.7 ± 10.1	–	–	1	6
Hans 2015	290	431	56	183	436	21	–	–	29		59	
John 2012	63	170	89	64	52	28	62	61	3	91	0	0
Joseph 2015	40	89	19	38	20	28	58 ± 9	56 ± 10	0	47	–	–
Malakh 2017	24	173	21	29	50	97	–	–	60		–	–
Maral 2018	45	135	–	–	–	–	75% EF ≥ 60%		4	71	–	–
Pietro 2012	24	108	–	–	–	–	88% EF > 45%		5		1	
Shunsuke 2020	414	589	–	–	–	–	–	–	–	–	73	
Suyog 2020	71	71	58	33	29	22	57 ± 5.4	57 ± 5.6	–	–	0	1

Author	BAV	TAV	Prolapse correction	
	(N)	(N)	BAV	TAV
(c)				
Aicher 2007	81	193	70	103
Carlos 2017	57	103	47	56
Dainius 2019	29	63	28	53
Hans 2015	290	431	–	–
John 2012	63	170	42	63
Joseph 2015	40	89	40	13
Malakh 2017	24	173	11	14
Maral 2018	45	135	42	59
Pietro 2012	24	108	10	3
Shunsuke 2020	414	589	–	–
Suyog 2020	71	71	–	–

### In-hospital mortality and reoperation rate

**Fig. 3:**
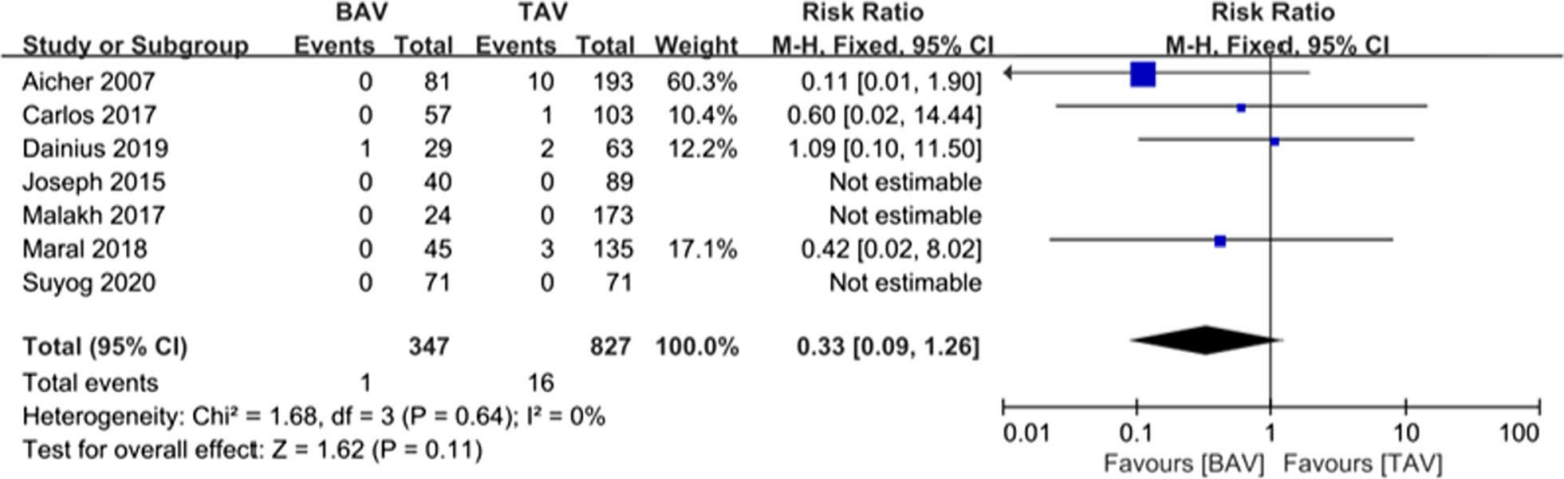
Forest plots of in-hospital mortality events of patients in BAV and TAV cohort

A total of 1174 patients were included in the in-hospital mortality analysis, of which 347 patients (29.6%) were in the BAV group, and 827 patients (70.4%) were in the TAV group. The in-hospital mortality rate was similar in both cohorts [0.00% vs. 1.93%; relative risk (RR) (95% confidence interval [CI]) 0.33 (0.09, 1.26), I^2^ = 0%, *P* = 0.11] (Fig. 3).

**Fig. 4:**
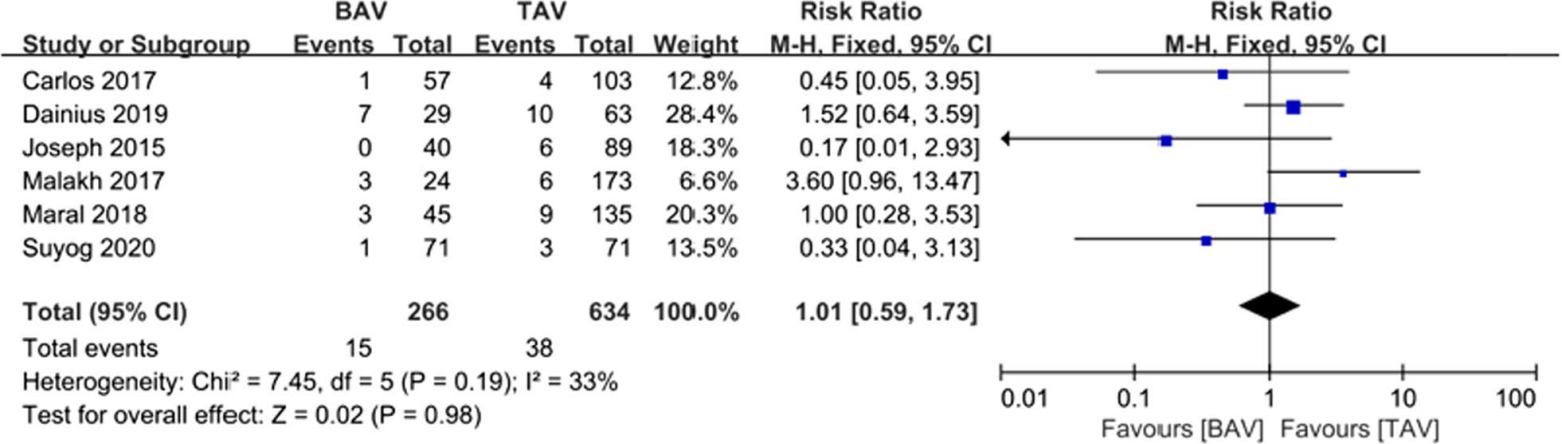
Forest plots of in-hospital reoperation events of patients in BAV and TAV cohort

The results for in-hospital reoperation were also comparable in the two groups. The TAV group showed slight but not significantly higher in-hospital reoperation rate [5.64% vs. 5.99%; RR (95% CI) 1.01(0.59, 1.73), I^2^ = 33%, *P* = 0.98] (Fig. 4). A total of 2198 patients were included in the in-hospital reoperation analysis; of these patients, 107 patients (4.9%) underwent reoperation, and most of them had the complication of bleeding.

### Mortality rate

**Fig. 5:**
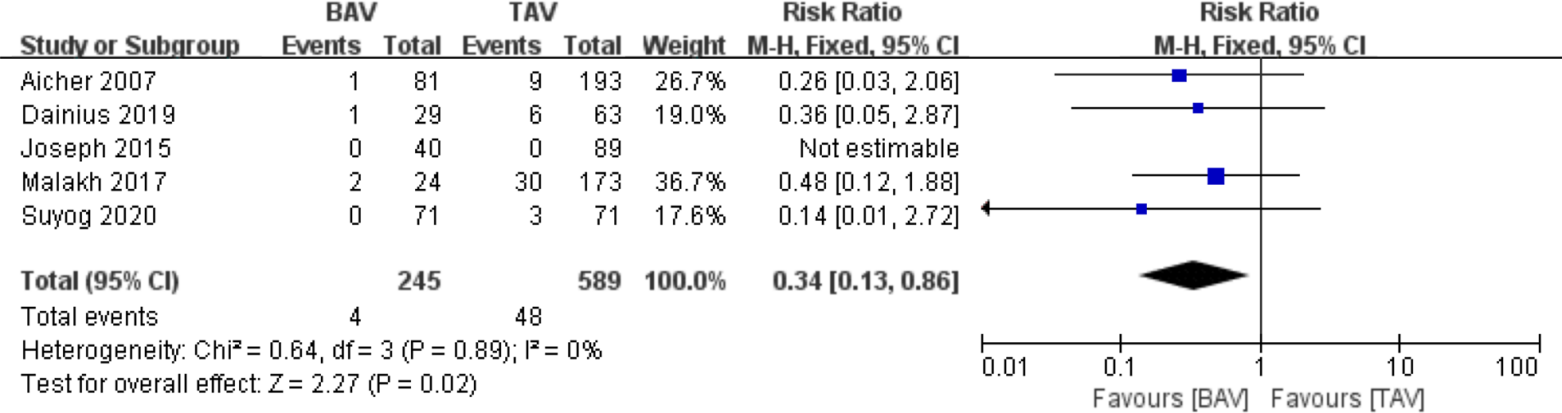
Forest plots of overall mortality events of patients in BAV and TAV cohort

Regarding the overall mortality rate (follow-up time is 5 years or more), BAV patients showed lower mortality rate than TAV patients [1.63% vs. 8.15%; RR (95% CI) 0.34(0.13, 0.86), I^2^ = 0%, *P* = 0.02] **(**Fig. 5**)**. A careful review of all the included studies revealed that the TAV group comprised more emergency cases, including acute aortic dissection. In the article of Aicher et al., the acute aortic dissection Stanford A(AADA) in TAV group is 40 patients, and only 6 AADA patients in the BAV group. The relatively high overall mortality rate in TAV group may have been influenced in part by patients with AADA.

### Reintervention rate

**Fig. 6:**
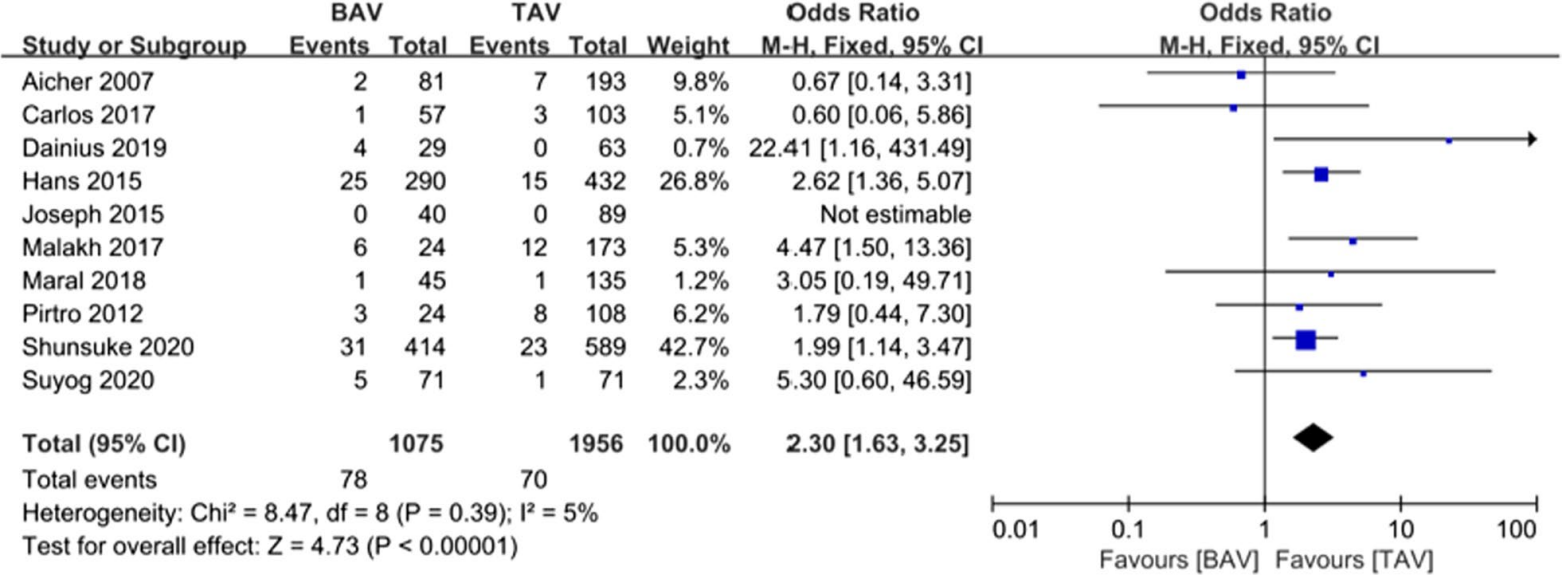
Forest plots of overall reintervention events of patients in BAV and TAV cohort

**Fig. 7:**
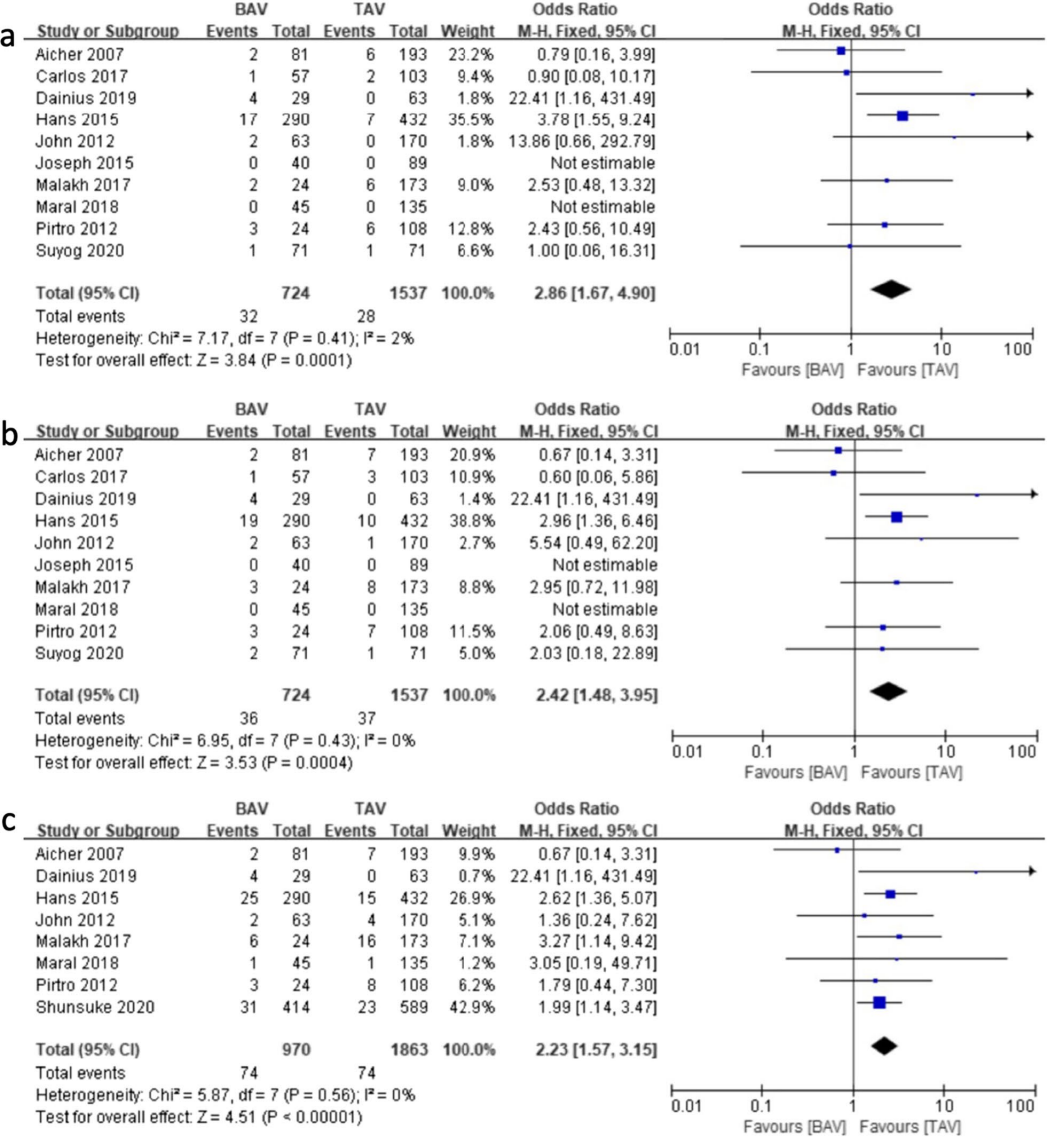
Forest plots of reintervention events of patients in BAV and TAV cohort in different follow-up periods. **a** Forest plots of short-term reintervention events of patients in BAV and TAV cohort. **b** Forest plots of mid-term reintervention events of patients in BAV and TAV cohort. **c** Forest plots of long-term reintervention events of patients in BAV and TAV cohort

Generally, over 1 year after the initial VSARR procedure, BAV patients were more likely to receive a second procedure due to recurrent aortic insufficiency [7.26% vs. 3.58%; odds ratio (OR) (95% CI) 2.36 (1.55, 3.60), I^2^ = 0%, *P* < 0.0001] (Fig. 6). To gain further insights, we analyzed the reintervention rate in different periods. As shown in Fig. 7a–c, short-term (within 3 years [4.42% vs. 1.82%; OR (95% CI) 2.86 (1.67, 4.90), I^2^ = 2%, *P* = 0.0001]), mid-term (within 5 years [4.97% vs. 2.41%; OR (95% CI) 2.42 (1.48,3.95), I^2^ = 0%, *P* = 0.0004]), and long-term (over 10 years [7.63% vs. 3.97%; OR (95% CI) 2.23 (1.57, 3.15), I^2^ = 0%, *P* < 0.0001]) reintervention rates were significantly higher in the BAV group.

**Fig. 8:**
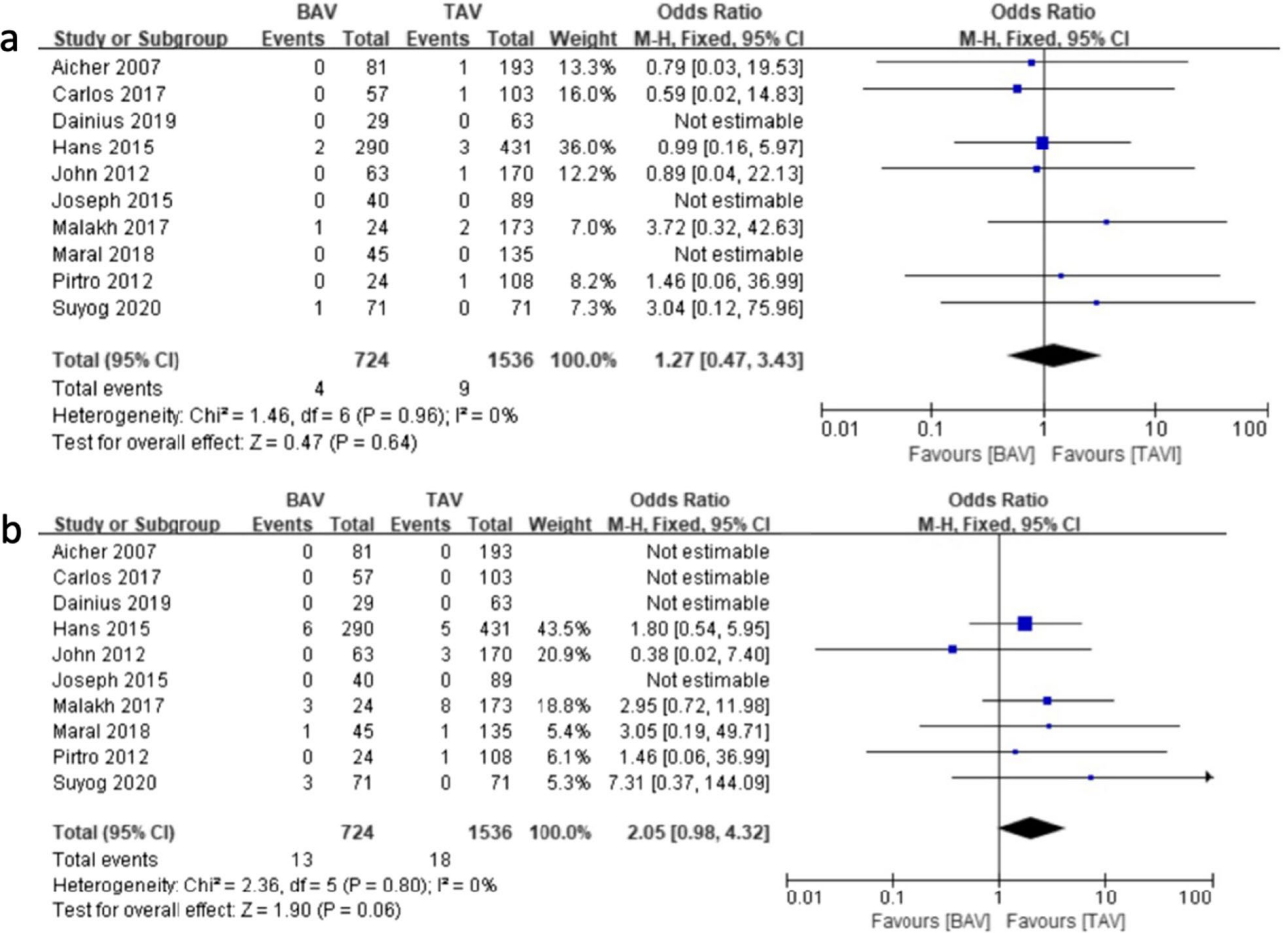
Forest plots of reintervention events of patients in BAV and TAV cohort in different follow-up periods. **a** Forest plots of reintervention events between 3 and 5 years follow-up of patients in BAV and TAV cohort. **b** Forest plots of reintervention events over 5 years of patients in BAV and TAV cohort

Surprisingly, after excluding the reintervention cases in the first three years, the reintervention rate between 3 and 5 years showed no significant difference between the two groups, four patients in BAV group were performed re-intervention and nine patients in TAV group. [0.55% vs. 0.59%; OR (95% CI) 1.27 (0.47, 3.43), I^2^ = 0%, *P* = 0.64] (Fig. 8a). We found that the TAV group showed a much lower reintervention rate after 5 years than the BAV group, but the difference was not significant. Total thirty-one patients underwent re-intervention after 5 years, of which 13 patients in BAV group and 18 patients in TAV group. [1.80% vs. 1.17%; OR (95% CI) 2.05 (0.98, 4.32), I^2^ = 0%, *P* = 0.06]) (Fig. 8b).

### Secondary endpoints

**Fig. 9:**
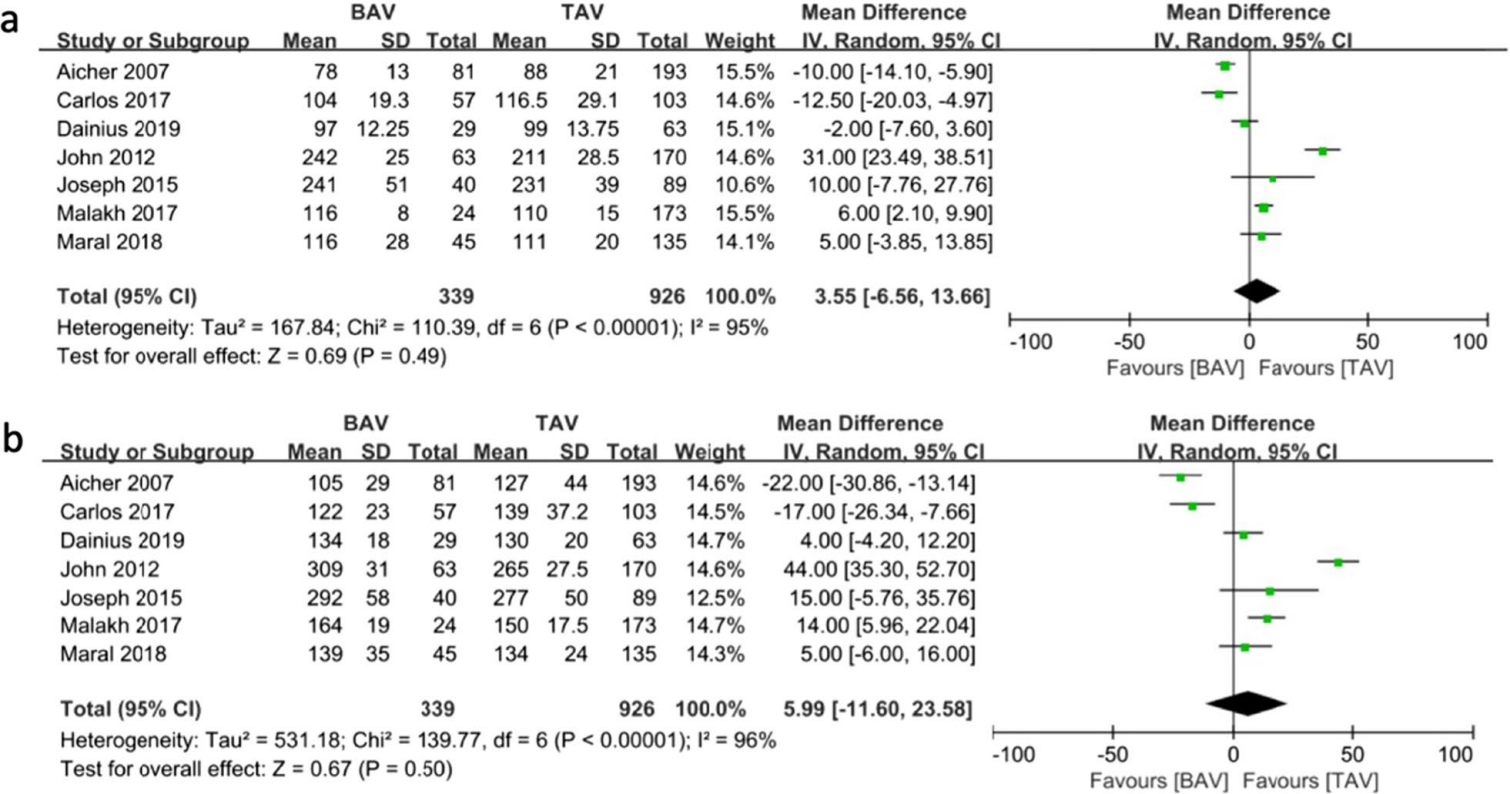
Forest plots of secondary endpoints of patients in BAV and TAV cohort in different follow-up periods. **a** Forest plots of aortic cross-clamping time of patients in BAV and TAV cohort. **b** Forest plots of total CPB time of patients in BAV and TAV cohort

The reported ACx seemed to be longer in patients with BAV than in patients with TAV; however, no significant difference was observed between the two groups (WMD: 3.55, 95% CI: [− 6.56, 13.66]; *P* = 0.49) **(**Fig. 9a**)**. The total CPB time was comparable in the two groups (WMD: 5.99, 95% CI: [− 11.60, 23.58]; *P* = 0.50) **(**Fig. 9b**)**.

## Discussion

**Fig. 10:**
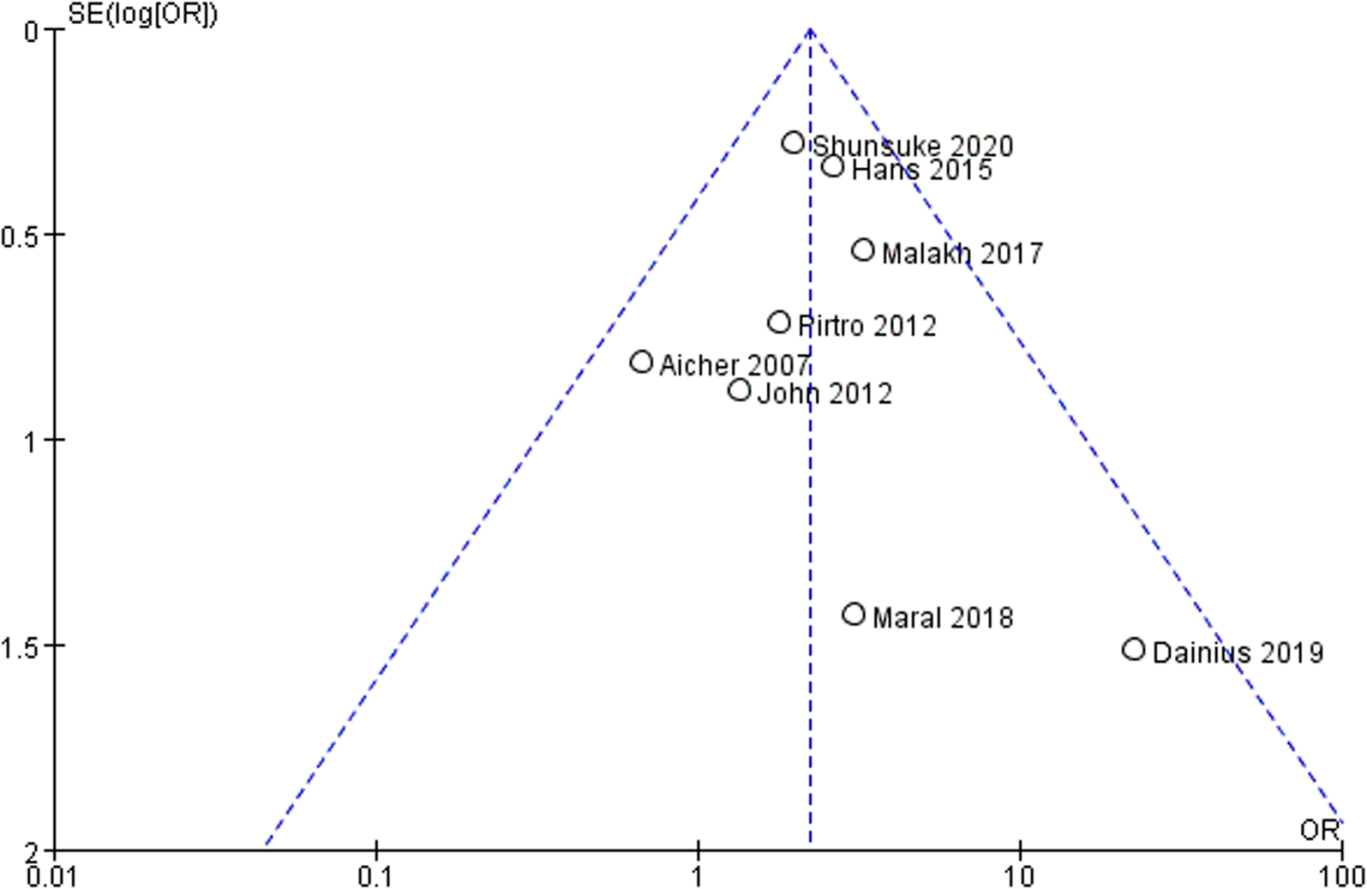
The funnel plot for included studies. The funnel plot was symmetrical which meant no significant publication bias. Compared long-term reintervention events of patients in BAV and TAV cohort

**Fig. 11:**
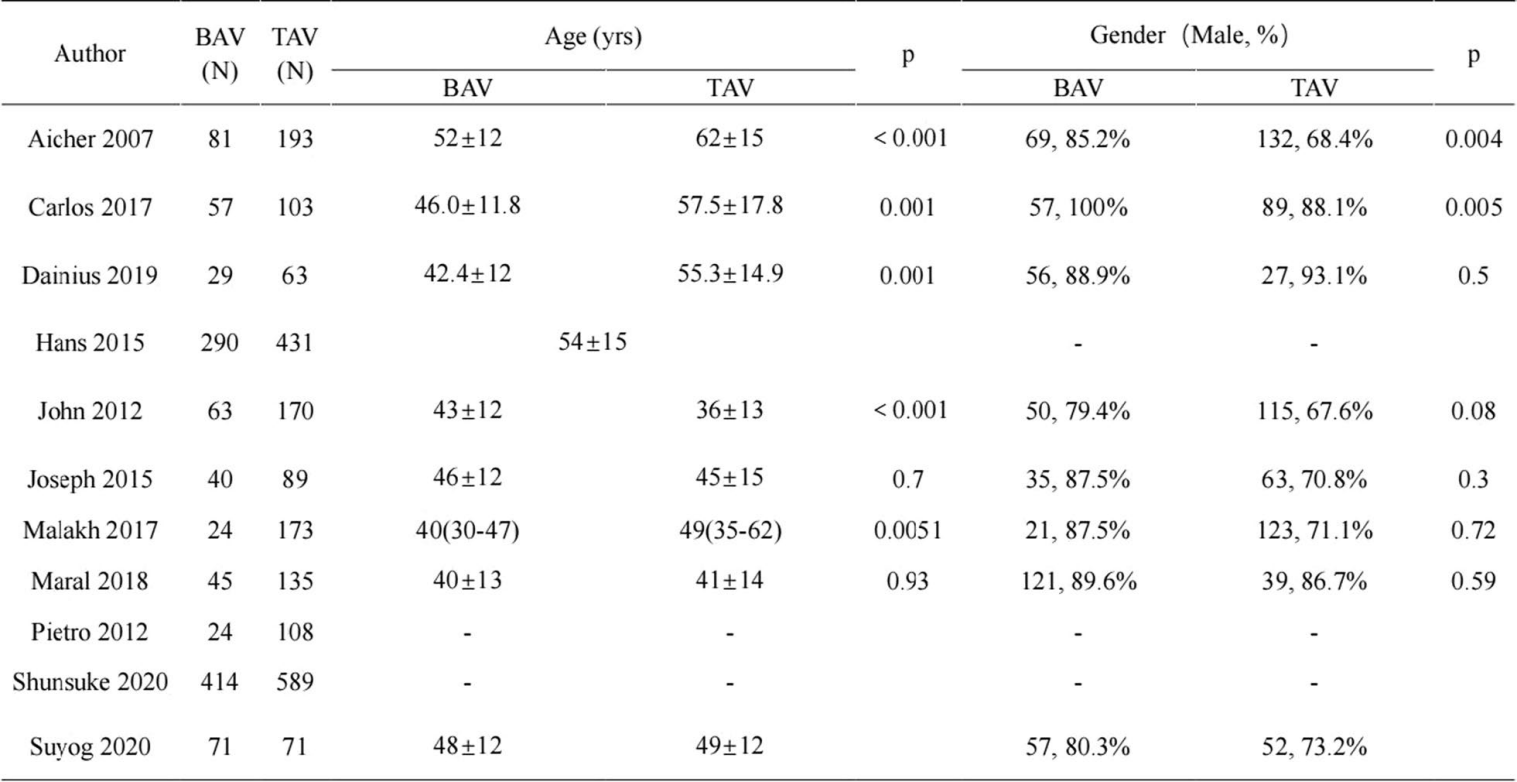
Summary of study characteristics

**Fig. 12:**
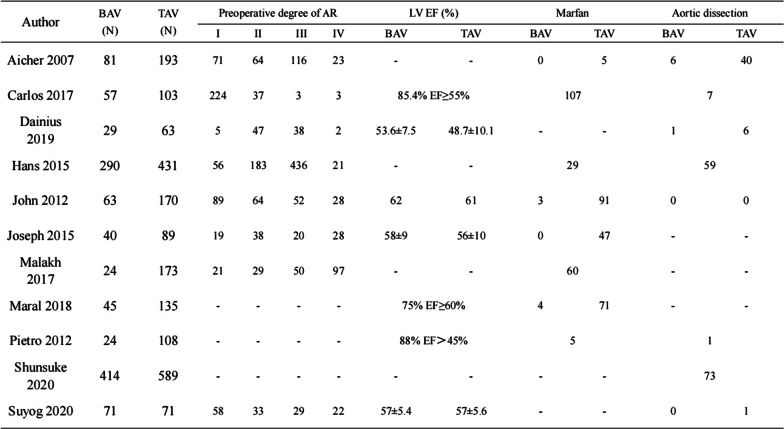
Summary of study characteristics

Two decades ago, the development of the VSARR procedure by Dr. Tirone David and Dr. Magdi Yacoub led to marked improvements in the outcomes of patients with aortic root dilation [20–23]. Both techniques yielded remarkable mid-term and long-term clinical results, and the choice of the surgical procedure mostly depended on the surgeon’s preference and expertise [21, 24]. Many studies have confirmed the safety and practicality of VSARR techniques [25]. It is well known that VSARR enables the patients to become free of anticoagulation-related bleeding and any possible future complications such as thromboembolism, stroke, and endocarditis [24]. Therefore, the VASRR techniques is a remarkable surgical procedure (Figs. 10, 11, 12).

Compared to its excellent and widely accepted clinical outcomes in patients with TAV, VSARR in patients with BAV remains controversial. Although several studies have demonstrated comparable short-term results of VSARR in patients with BAV, some studies have raised concerns regarding recurrent aortic regurgitation and progressive aortic stenosis [26]. Notably, in a series of 108 consecutive patients who underwent isolated BAV repair, the reintervention rate was 51% at a 10-year follow-up [27]. Svensson et al. reported the long-term durability of BAV repair. Freedom from aortic reintervention was determined to be 87%, 78%, and 64% at 5, 10, and 15 years, respectively. The most common reason was cusp prolapse, and aortic regurgitation from root aneurysm was noted in 15% of the cases [28]. Most recently, Kalra et al. illustrated the safety and usefulness of VSARR in patients with BAV deformity [29].

In the present study, the perioperative data between patients with TAV and BAV were analyzed. Regarding safety concerns of the procedure, the rates of in-hospital mortality and reoperation due to bleeding were comparable between the two groups. Consistent with most other studies, VSARR for patients with BAV and TAV can be considered a safe alternative method [2, 30].

Many studies have reported that patients with BAV who underwent VSARR procedure had a higher incidence of leaflet repair, including plication of the free edges, free edge reinforcement, triangular resection, or a combination of these techniques [31]. In our included studies, many studies emphasized the higher incidence of leaflet repair and longer ACx. However, we found no difference between the two groups regarding the total ACx and CPB time. A careful review of all studies showed that patients with acute aortic dissection were also enrolled in many studies, but exclusively or mostly, these patients were present in the TAV group. We also found that the concomitant CABG rate was significantly higher in the TAV group, especially in Dr. Aicher’s study [11]. Moreover, in Dr. Aicher’s study, all concomitant CABG procedures were performed because of preoperative coronary artery disease instead of intraoperative coronary injury or myocardial infarction. Therefore, the baseline between the two groups was not balanced. When encountering patients with TAV, surgeons seem to be more confident and willing to challenge the established guidelines for treatment.

In our analysis, all-cause mortality over 1 year was significantly higher in the TAV group. Although there is no further explanation regarding this aspect in the respective studies but according to our analysis, there are several reasons for this result. First, because of the large number of patients in the TAV group, which was twice as large as that in the BAV group, some patients died from non-cardiovascular causes. The second reason is that acute aortic dissection is more common in the TAV group, which also increases the mortality rate in the TAV group. Third, the average age of BAV patients in many of the reported cases was younger and the TAV group patients had more morbidities.

The long-term durability of the aortic valve is the most important factor considered after the perioperative period. Short-term and mid-term (in 5 years) outcomes of VSARR were reported to be comparable in both TAV and BAV groups [30]. However, given the long-term results of VSARR, it was unclear whether the valve durability was still comparable in the BAV and TAV groups. In our study, we found a significantly higher incidence of reintervention in the BAV group (*P* < 0.00001). Further review of the included studies revealed that most of the reoperations were performed due to recurrent aortic regurgitation and other reasons, including aortic stenosis and endocarditis. More importantly, cusp prolapse was mostly responsible for recurrent aortic regurgitation. Many factors might increase the risk of reoperation, including large aortoventricular diameter, use of a pericardial patch, and less effective height. Dr. Schafers first described the concept of effective height in 2007 and the application of aortic annuloplasty in 2009; following these reports, valve durability after the remodeling procedure was significantly improved [32, 33]. Especially in patients with BAV, annuloplasty is a substantial element of repair due to the inherent annulus enlargement (annular ectasia) [34]. As mentioned above, cusp plasty was performed considerably more often in the BAV group, which indicated a higher rate of morphological alteration of BAV cusps at the time of operation.

Regarding progressive aortic stenosis, the post-operative mean gradient of BAV was reported to be slightly higher than that of TAV [14]. Vallabhajosyula et al. reported that one patient had a post-operative discharge peak gradient of > 20 mm Hg and was free from any reported effects of aortic stenosis [35]. Therefore, although patients with BAV have a slightly higher transvalvular gradient, it is unlikely to have any clinical relevance and relation to the development of severe aortic stenosis.

Freedom from aortic reintervention is always an essential factor in the valve-sparing procedure. In our analyses, we found that most reinterventions occurred in the first 3 years, and a significantly higher incidence of reinterventions was noted in the BAV group. Although all short-term, mid-term, and long-term outcomes showed significant differences between the BAV and TAV groups, the difference was no longer significant after excluding these reinterventions that occurred in the first 3 years. Which means, the long-term reintervention rate (5 years or more), TAV group showed a slight but no statistically advantage. According to the present data, we infer, if the population size was larger, the advantage in TAV group was greater. And we carefully interpreted that patients with BAV who underwent VSARR might go through both short-term and long-term risk periods.

Many studies showed comparable findings between VSARR and Bentall procedure with a bioprosthesis [29, 36]. Dr. Kalra et al. reported an equivalent 10-year reintervention incidence in patients with BAV who underwent VSARR or Bentall procedure. More recently, a large cohort study compared the 10-year freedom from aortic reintervention between the VSARR group and the bioprosthetic Bentall group and showed similar results between both the groups. In the present study, we found a higher aortic reintervention rate of 7.6% in patients with BAV, which is still comparable to those with bioprosthetic Bentall procedure (10.6%) [37].

## Limitation

The present meta-analysis has some limitations. First, single-arm observational studies that reported data only for BAV or TAV outcomes without any comparison were excluded. Second, even though we followed the ROBSIN-I guidelines to evaluate the risk of bias in the included studies, there were several possible confounding factors. Third, not all the studies included in the analysis had the same variables used for propensity matching or for Cox hazard grouping. Fourth, regarding the long-term mortality rate, because of the limitation of the original texts and other content included, the results obtained were exactly the all-cause mortality rate after the initial VSARR. Because it was impossible to extract and determine whether the cause of death in long-term mortality patients in each article was related to the heart or aorta, subsequent cohort studies with larger sample size and a longer follow-up time may be required to confirm this aspect. Fifth, limited observational data were available in both BAV and TAV groups to determine outcomes that are predicted with a high degree of sensitivity and specificity.

## Conclusion

VSARR for treating the dilated aortic root is an attractive surgical approach for patients with either TAV or BAV. The safety and short-to mid-term effectiveness of VSARR were fully established by many well-designed studies. Regarding long-term results, the present data showed less valve durability in patients with BAV than in patients with TAV. However, the optimally selected patients with BAV (requiring less cusp plasty and post-operative high effective height) may still gain maximal benefits from VSARR.

## Data Availability

All data generated or analyzed during this study are included in this published article and its supplementary information files.
